# Lower Recovery of Nontuberculous Mycobacteria from Outdoor Hawai’i Environmental Water Biofilms Compared to Indoor Samples

**DOI:** 10.3390/microorganisms9020224

**Published:** 2021-01-22

**Authors:** Ravleen Virdi, Melissa E. Lowe, Grant J. Norton, Stephanie N. Dawrs, Nabeeh A. Hasan, L. Elaine Epperson, Cody M. Glickman, Edward D. Chan, Michael Strong, James L. Crooks, Jennifer R. Honda

**Affiliations:** 1 Center for Genes, Environment, and Health, National Jewish Health, Denver, CO 80206, USA; ravleen.virdi@hotmail.com (R.V.); grantn78@gmail.com (G.J.N.); DawrsS@njhealth.org (S.N.D.); HasanN@njhealth.org (N.A.H.); EppersonE@njhealth.org (L.E.E.); StrongM@njhealth.org (M.S.); 2 Division of Biostatistics and Bioinformatics, National Jewish Health, Denver, CO 80206, USA; melowe1216@gmail.com (M.E.L.); CrooksJ@njhealth.org (J.L.C.); 3 Department of Biostatistics and Informatics, Colorado School of Public Health, Aurora, CO 80045, USA; 4 Computational Biosciences, University of Colorado Anschutz Medical Campus, Aurora, CO 80045, USA; CODY.GLICKMAN@cuanschutz.edu; 5 Department of Medicine and Academic Affairs, National Jewish Health, Denver, CO 80206, USA; ChanE@njhealth.org; 6 Division of Pulmonary Science and Critical Care Medicine, University of Colorado Anschutz Medical Campus, Aurora, CO 80045, USA; 7 Department of Medicine, Rocky Mountain Regional Veterans Affairs Medical Center, Denver, CO 80523, USA

**Keywords:** nontuberculous mycobacteria, biofilms, environment, Hawai’i

## Abstract

Nontuberculous mycobacteria (NTM) are environmental organisms that can cause opportunistic pulmonary disease with species diversity showing significant regional variation. In the United States, Hawai’i shows the highest rate of NTM pulmonary disease. The need for improved understanding of NTM reservoirs led us to identify NTM from patient respiratory specimens and compare NTM diversity between outdoor and indoor locations in Hawai’i. A total of 545 water biofilm samples were collected from 357 unique locations across Kaua’i (*n* = 51), O’ahu (*n* = 202), Maui (*n* = 159), and Hawai’i Island (*n* = 133) and divided into outdoor (*n* = 179) or indoor (*n* = 366) categories. *rpoB* sequence analysis was used to determine NTM species and predictive modeling applied to develop NTM risk maps based on geographic characteristics between environments. *M. chimaera* was frequently identified from respiratory and environmental samples followed by *M. chelonae* and *M. abscessus;* yet significantly less NTM were consistently recovered from outdoor compared to indoor biofilms, as exemplified by showerhead biofilm samples. While the frequency of *M. chimaera* recovery was comparable between outdoor and indoor showerhead biofilms, phylogenetic analyses demonstrate similar *rpoB* gene sequences between all showerhead and respiratory *M. chimaera* isolates, supporting outdoor and indoor environments as possible sources for pulmonary *M. chimaera* infections.

## 1. Introduction

Nontuberculous mycobacteria (NTM) are environmentally acquired opportunistic pathogens that may cause refractory pulmonary disease (PD). Recent reports indicate that NTM pulmonary infections are associated with a 4.3-fold higher incidence of chronic respiratory failure and increased mortality rates when compared to the general population [1,2]. The five-year mortality rate was found to be 35% in a study of 316 patients with NTM respiratory isolates [2]. Additionally, patients with NTM PD were 40% more likely to succumb to their disease over a 10-year study period than patients without NTM PD [3]. The most common NTM pathogens responsible for NTM PD in adults include species that comprise the *Mycobacterium avium* complex (MAC) and *Mycobacterium abscessus* [4]. The urgent need to understand where patients acquire their infections and how to prevent the initial infection drives the need for more studies that identify the environmental niches for NTM in high-risk geographic areas.

In the United States (U.S.), Hawai’i is a geographic hot spot for NTM, hosting the highest prevalence of pulmonary NTM infections with an 11-year period prevalence nearly four times higher than the total U.S. population [5]. NTM infections are difficult to treat and thought to be environmentally acquired from the inhalation of NTM-laden aerosols generated from freshwater biofilms and soil. Through molecular and phylogenetic comparisons of NTM isolated from non-NTM patient household plumbing biofilms and soil as well as a small number of patient respiratory specimens, we previously reported that *Mycobacterium chimaera* (now classified as *M. intracellulare* subsp. *chimaera*) [6,7], a member of the MAC, was the dominant NTM in both households and clinical sample types [8], and *Mycobacterium avium* was notably absent from the environmental samples tested [8]. In the same study, *M. abscessus* subsp. *abscessus* was prevalent in kitchen sink and bathroom sink/showerhead biofilms, but absent from soil samples tested, while the MAC species *Mycobacterium intracellulare* was identified from a single soil sample. These data suggest a preponderance of clinically relevant NTM in Hawai’i household plumbing.

In the current study, NTM was identified from an expanded collection of clinical respiratory specimens from Hawai’i patients with suspected mycobacterial lung infections. In parallel, environmental sampling in Hawai’i was broadened to include samples from non-household locations, including freshwater biofilms from sites located outdoors in addition to freshwater streams and lava rocks as comparisons to indoor (e.g., household) water biofilm samples. Because *M. chelonae* has been recovered from sphagnum moss and soil rich in sphagnum and peat moss [9,10,11], naturally growing sphagnum moss and green peat were also sampled. Furthermore, we developed a predictive model using geographic characteristics across the islands to estimate the relative risk of positive NTM culture among outdoor and indoor environments. Delineation of the environmental reservoirs for these emerging pathogens may provide avenues of interests dedicated to uncovering new potential intervening strategies for NTM control and prevention within the environment, particularly in geographic areas of high disease burden.

## 2. Materials and Methods

### 2.1. Clinical Respiratory NTM Isolates

Similar to our prior work [8], NTM respiratory isolates were recovered from 218 de-identified Hawai’i patients with suspected tuberculosis whose sputum had been submitted for mycobacterial culture and processed by the Diagnostic Laboratory Services, Inc. (Aiea, HI). After tuberculosis testing by mycobacterial growth indicator tubes, leftover samples were randomly selected from an archival library and sub-cultured into Middlebrook 7H10 slants. Random selection was accomplished by using an electronic random number generating table. *Mycobacterium tuberculosis* was not recovered in any of the respiratory disease cases where NTM was isolated. Specific details about these patients were not available at the time, so it is not known if these patients met current American Thoracic Society/Infectious Disease Society of America (ATS/IDSA) diagnostic criteria for NTM PD [12].

### 2.2. Environmental NTM Sampling

A total of 373 unique environmental sites were sampled across Kaua’i, O’ahu, Maui, and Hawai’i Island. A total of 545 water biofilm swabs were collected from these unique sites, of which 179 samples were collected from various outdoor sites. The remaining 366 samples were collected from indoor sites. The sample distribution and the total number of samples collected are detailed in Table 1.

Biofilm samples were collected using sterile synthetic flock dual-tipped swab applicators (Puritan HydraFlock Sterile Flocked Collection Devices #25-3306 2HBT, Guilford ME). Outdoor samples were collected by swabbing the surface of publicly accessed beach showerheads, sides of swimming pools at the air-water interface, the inner surface of dispensers from outdoor ice machines, inside the spouts of public water fountains, as well as lava rocks, large rocks found in natural streams at the water-air interface, and the surface of sphagnum moss. Indoor water biofilm swab samples were obtained by swabbing surfaces at the inner surface of showerheads, kitchen, and bathroom faucets from volunteer households as we have previously published [8].

### 2.3. Recovery of NTM from Environmental Samples

Standard microbiological approaches were used for the isolation of environmental NTM [8]. In brief, swabs were immersed in two mL of autoclaved ultrapure water and vortexed on high for one minute. Next, 450 µL of sample was transferred to a sterile tube with 50 µL of 1% cetylpyridinium chloride (CPC), vortexed for one minute on high, and incubated at room temperature for 30 min [13]. After incubation, the sample was vortexed and 100 µL was spread-plated on duplicate Middlebrook 7H10 agar plates with oleic acid/glycerol enrichment. One plate was incubated at 30 °C and the other at 37 °C for three weeks. Following incubation, colonies were picked and grown in supplemented Middlebrook 7H9 broth for the isolation of genomic DNA. One mL of culture was centrifuged at 1300× *g* for one minute at room temperature after which the supernatant was discarded and the bacterial pellet stored at −80 °C to be used for NTM identification.

### 2.4. NTM DNA Isolation

Intact genomic DNA was extracted from bacterial pellets according to Epperson and Strong [14] including the optional bead-beating step.

### 2.5. NTM Species and Subspecies Identification by Partial rpoB Gene Sequencing

Identification of environmental NTM isolates was conducted through the amplification and sequencing of an ~ 700 bp segment of the RNA polymerase Region Five beta subunit (*rpoB*) gene [15,16]. PCR reactions included 1–10 ng of template DNA, 2 μL each of 5 μM forward and reverse *rpoB* primers, and nuclease-free water combined in 96 well plates which were submitted for Sanger sequencing (Quintara Biosciences, San Francisco, CA, USA). Sequences were trimmed for quality and compared against *rpoB* type strain sequences deposited in the National Center for Biotechnology Information (NCBI) GenBank using the BLAST algorithm. Samples with *rpoB* amplicon sequences aligning with uncultured mycobacterial species were deemed to be novel species.

### 2.6. Geospatial Modeling of NTM in Hawai’i through Machine Learning

Of the 357 locations sampled in this study, 350 were used to develop models based on the completeness of geospatial characteristics. After NTM species identification, machine learning tools were applied to correlate microbiological culture information to geospatial variables in order to generate predictive maps of the probability of finding NTM at unsampled locations across the islands. GIS (geographic information system) data on the Hawaiian Islands were retrieved including climatological and socio-economic variables from the U.S. Census and American Community Survey, U.S. Geological Survey, The State of Hawai’i, and the University Hawai’i, Mānoa. These GIS layers were evaluated for the outdoor and indoor sample locations, including those samples that yielded either a positive or negative NTM culture results. Geospatial variables were included in the model based on the completeness of the GIS data layer for each candidate variable.

To map the probability of NTM presence, a grid with 1 km^2^ resolution was created over the islands consisting of 15,843 locations and the geospatial layers at those locations evaluated. Smaller islands without NTM sampling such as Kaho’olawe, Moloka’i, Lana’i, and Ni’ihau were not included. The spatial range of the locations was limited to altitudes above sea level, but lower than the maximum elevation documented by NTM sampling.

Predictive maps were created using an “eXtreme Gradient Boosting” machine learning algorithm from the caret package 6.0–85 in R version 3.6.0 [17,18], which predicts the probability of NTM presence at each grid location given the geospatial and NTM data at the sample locations. Note that machine learning algorithms of this type (tree-based methods) are designed to generate accurate predictions, but do not yield estimates of linear association between predictors and outcomes and in fact explicitly assume nonlinear associations. Ten-fold cross-validation with 100 repeats was used to evaluate which geospatial variables were most important for predicting NTM presence.

### 2.7. Showerhead and Clinical Mycobacterium chimaera Phylogenetic Comparisons

For a subset of showerhead and clinical *M. chimaera* isolates, partial *rpoB* gene sequences were aligned using MUSCLE [19]. A neighbor-joining phylogenetic tree was estimated from realigned *rpoB* sequences to examine *rpoB* sequence variation based on the number of nucleotide differences and uniform rates among sites, while omitting any sites in the alignment with gaps or missing data between showerhead and clinical isolates [20]. The phylogenetic analysis included 100 bootstrap replicates to represent the topology of the phylogenetic trees which were visualized using ggtree [21]. SNP variation between all *M. chimaera* isolates in this study are summarized in Table 2.

### 2.8. Statistical Analyses

The data were analyzed with GraphPad Prism 8.4.3. Fisher’s Exact Tests were used to evaluate differences in proportions of NTM species or species groups between outdoor and indoor samples.

## 3. Results

### 3.1. M. chimaera Predominates Hawai’i Respiratory Samples

**Figure 1:**
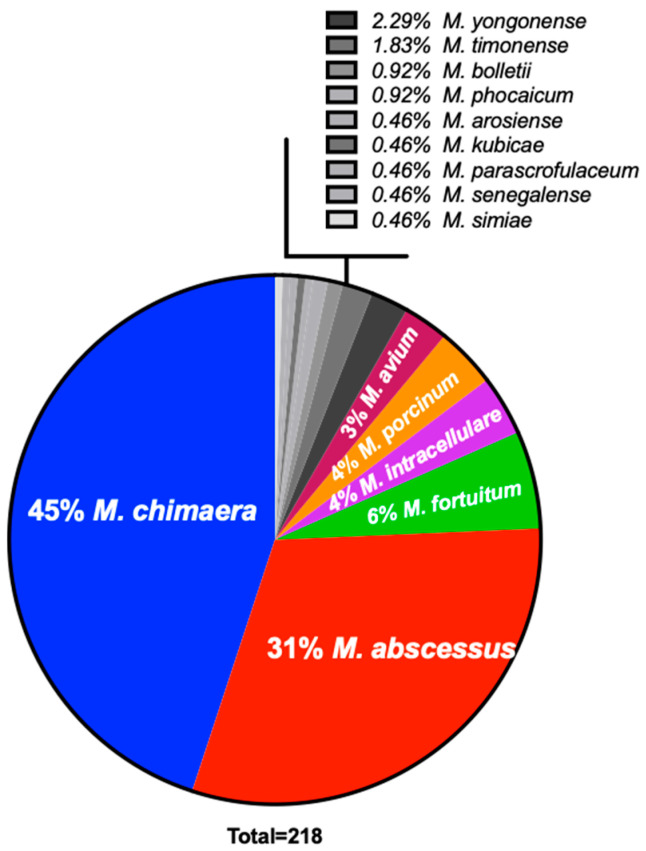
*M. chimaera* predominates clinical respiratory specimens from Hawai’i. NTM respiratory isolates were obtained from 218 de-identified Hawai’i patients with suspected mycobacterial lung infection. *M. chimaera* was the most commonly identified, followed by *M. abscessus, M. porcinum, M. fortuitum, M. intracellulare* and *M. avium.*

From 218 de-identified respiratory patient samples with suspected mycobacterial lung infections in Hawai’i (Figure 1), *M. chimaera* was most commonly isolated (45%), followed by *M. abscessus* (31%), *Mycobacterium fortuitum* (6%), and *Mycobacterium porcinum* (4%). In contrast, *M. intracellulare* (4%) and *M. avium* (3%) were found much less frequently among this group of respiratory samples.

### 3.2. Geospatial Variables Associated with NTM between Outdoor and Indoor Samples

**Figure 2:**
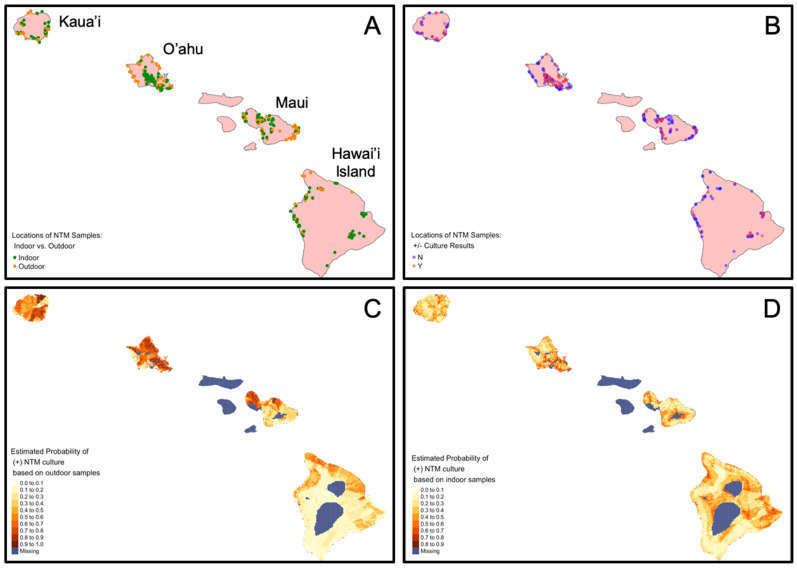
Sample locations stratified by outdoor and indoor locations, NTM microbiological culture results, and probability of NTM detection based on predictive models. The same sampled sites are shown on each map representing all sample locations for this study, which were also used in predictive modeling. (**A**) Map identifies the samples collected from outdoor (orange) or indoor (green) sources. (**B**) Map identifies the sample locations that were NTM culture-positive (“Y, red dots) or NTM culture-negative (“N”, blue dots). Dark dots indicate locations where sample collection sites overlap. Map (**C**) indicates the probability of NTM in outdoor locations based on 134 outdoor samples. Accuracy estimate: 0.70. Map (**D**) indicates the probability of NTM in indoor locations based on 216 indoor samples. Accuracy estimate: 0.56.

Outdoor and indoor collection site locations and their stratification by NTM culture results are shown in Figure 2A,B. Machine learning tools were subsequently applied to associate NTM culture status with geospatial variables in predictive models. The predictive models were then applied to 1 km^2^ grid of points across the islands to develop risk maps of positive NTM culture for outdoor and indoor samples. The risk map showing the probability of finding NTM in outdoor locations is shown in Figure 2C and the risk map showing the probability of finding NTM in indoor locations is shown in Figure 2D. The positive predictive accuracy of the outdoor model was 0.70 with a Cohen’s kappa value of 0.23. The positive predictive accuracy of the indoor model was lower at 0.56 with a Cohen’s kappa of 0.1, suggesting that the outdoor model predicts where a positive culture would occur 70% of the time relative to the 50% associated with random chance.

The geospatial variables of the highest importance in predicting a positive NTM culture varied depending on whether the NTM was recovered from an outdoor or indoor sample. The full list of geospatial variables associated with NTM in both outdoor and indoor samples are tabulated in Table 3. We include the variable rankings for both outdoor and indoor models regardless of the model’s predictive ability. NTM culture-positive samples from outdoor locations were most influenced by elevation, lowest income quintile, the total population in the census tract, watershed area, and population of Pacific Islanders with a probability of correct prediction at 0.70. Indoor location based models were influenced by average annual evapotranspiration, elevation, average annual rainfall, median gross rent, and the GINI coefficient (a measure of income inequality), but were only slightly better than a coin-flip (probability = 0.56) at pinpointing where NTM might be found. While the model indicates the importance of each variable, it does not indicate directionality or linearity. NTM species identification seemed to be an important characteristic that varied between outdoor and indoor samples, but there was inadequate power to evaluate differences in species type. We emphasize that these variables do not necessarily indicate causality. The possibly unknown true causal factor(s) driving NTM presence may simply be correlated with the variables in our model.

### 3.3. Outdoor Water Biofilm Samples Show Significantly Less NTM Compared to Indoor Water Biofilm Samples

Of the 357 unique sites sampled across four islands, NTM was recovered from 42.9% (153/357). From these locations, more than one swab may have been collected (e.g., a showerhead and kitchen sink biofilm sample may have been collected from the same household site). In total, 545 total water biofilm samples were procured and NTM was isolated from 33.8% (184/545) of these total samples. Table 4 shows the percent positivity rate of NTM culture between outdoor and indoor samples collected across Kaua’i, O’ahu, Maui, and Hawai’i Island. While there was no significant difference in NTM recovery between outdoor and indoor samples collected from Kaua’i, Maui, and Hawai’i Island, the outdoor samples from O’ahu showed significantly less NTM compared to indoor samples from Oahu (****, *p* < 0.0001). More broadly, across all islands, significantly less of the total number of outdoor samples were NTM culture positive 40/179 (22.3%) compared to the total number of indoor biofilm samples tested (150/366, (41.2%) (****, *p* < 0.0001).

### 3.4. NTM Species Diversity and Recovery Proportions Vary by Island

**Figure 3:**
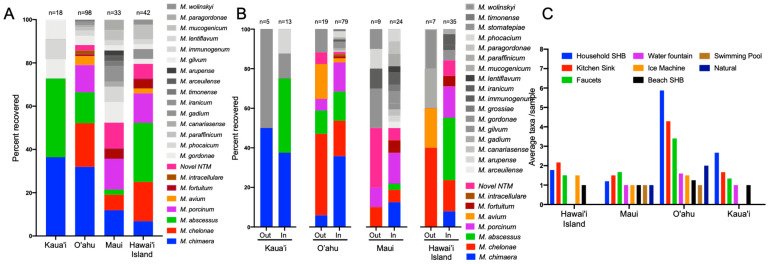
Clinically relevant NTM species predominate in Hawai’i environmental samples. (**A**) Colored bars correspond to the species of NTM identified using *rpoB* gene sequencing from all water biofilm samples stratified by island. Isolates shown in greyscale are generally not associated with pulmonary disease. The total number of NTM isolates recovered per sample type is shown above each column. (**B**) Data shown in “A” stratified by island and collection location (e.g., indoor or outdoor). The total number of NTM isolates recovered per sample type is shown above each column. (**C**) Average number of NTM species recovered per sample type per island as a measure to assess NTM diversity across different sample types. SHB = showerhead biofilm. Faucets include all bathroom and public restroom sink faucets.

To investigate the significance of NTM species diversity across the sampled islands, *M. chimaera, M. chelonae, M. abscessus*, *M. porcinum, M. avium,* and *M. intracellulare* were categorized as clinically relevant NTM species and denoted by the colored bars shown in Figure 3A,B, while species less associated with causing *bone fide* NTM PD (e.g., *M. gadium* [22], *M. canariasense* [23], and others) were represented using greyscale bars in the same figures. Clinically relevant NTM were recovered from all four islands tested, but 87% of the 98 Oahu samples that were NTM culture positive were clinically relevant species compared to 50% of the Maui samples (Figure 3A). Although a small number of the Kaua’i samples were NTM culture positive (*n* = 18), *M. abscessus* and *M. chimaera* were equally represented (36%, respectively). *M. chimaera* was the most frequently recovered NTM from O’ahu water biofilm samples (36%), followed by *M. chelonae* and *M. abscessus*. *M. porcinum* was the most common NTM identified from Maui (14%), while *M. abscessus* was the most frequently identified NTM among the environmental samples from Hawai’i Island (27%) (Figure 3A).

To determine whether NTM recovery and diversity differed between outdoor and indoor locations, these features were stratified by island (Figure 3B). For both Kaua’i and Hawai’i Island, clinically relevant NTM species were more closely associated with indoor samples compared to the outdoor samples collected on those islands. In contrast, the recovery of PD-causing NTM species such as *M. chimaera, M. chelonae, M. abscessus*, and *M. porcinum* represented more than 80% of the NTM identified in all samples from O’ahu. In contrast, within the Maui samples, these species were equally represented (~ 50%) among the outdoor and indoor samples. For the first time, *M. avium* was identified from Hawai’i environmental samples tested, comprising 17.6% and 20.0% of the outdoor samples from O’ahu and Hawai’i Island respectively, and 2.1% of indoor samples from O’ahu. Additionally, *M. intracellulare* was rarely recovered from the Hawai’i environment, representing 1% of indoor samples from O’ahu and was not found on any of the other islands sampled.

Of the NTM identified, indoor water biofilms including those collected from household showerheads, kitchen sinks, and bathroom faucets were common niches for NTM, particularly on O’ahu, with an average of six, four, and three NTM species identified in these respective sample types (Figure 3C). Of the eight types of different water biofilm samples collected (i.e., household showerheads, kitchen sinks, bathroom faucets, water fountains, ice machines, beach showerheads, swimming pools, and natural sites), NTM were recovered from all eight types on O’ahu and varied in recovery among the other island samples.

### 3.5. Low Recovery of NTM from Natural Environments of Hawai’i

**Figure 4:**
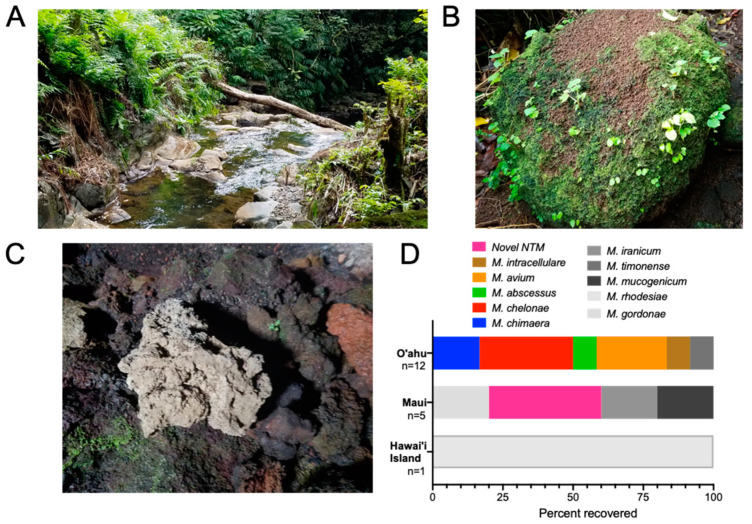
Natural areas of Hawai’i do not harbor significant NTM. (**A**) Representative image of freshwater streams that were sampled for NTM. In these locations, large rocks or boulders lodged in the freshwater stream were sampled at the air-water interface. (**B**) Representative image of moss that were typically located growing on the surface of large boulders. (**C**) Representative image of lava rocks sampled. Swabs were wiped across the surface of rocks and in cervices although these surfaces were devoid of NTM. (**D**) Diversity of NTM species recovered from stream-rock interface biofilms and moss from O’ahu, Maui, and Hawai’i Island.

While indoor water biofilms in Hawai’i clearly harbor NTM, their prevalence in the natural, outdoor environments of Hawai’i is less understood. To explore this, 61 samples were collected from a variety of natural locations by sampling rock boulders lodged in freshwater streams at the air-water interface (*n* = 28) (Figure 4A), the surface of sphagnum moss typically found atop of stream rocks or large boulders (*n* = 26) (Figure 4B), and the surface of lava rocks (*n* = 7) (Figure 4C). Of these 61 samples, 19.7% (*n* = 12) were NTM culture positive. From the 13 natural samples collected from Hawai’i Island, one was NTM culture positive and identified as *M. rhodesiae* from a moss sample (Figure 4D, Table 5a, b). Of the Maui samples, a novel isolate of NTM was recovered from one of the moss samples and a freshwater stream sample. However, a different freshwater stream sample contained a combination of NTM species including *M. gordonae, M. iranicum*, and *M. mucogenicum* (Table 5a,b). Most of the aforementioned NTM species are not typically associated with PD. In comparison, 8/10 (80%) of the freshwater streams on O’ahu harbored a variety of clinically relevant NTM including *M. chimaera, M. abscessus, M. avium*, and *M. intracellulare* (Figure 4D, Table 5a,b). In general, the moss and lava rocks sampled across all islands were not common sources of NTM.

### 3.6. Significantly Less NTM from Outdoor Beach Showerhead Biofilms (SHB) Compared to Indoor SHB, but Equal Possibility of M. chimaera Exposure

**Figure 5:**
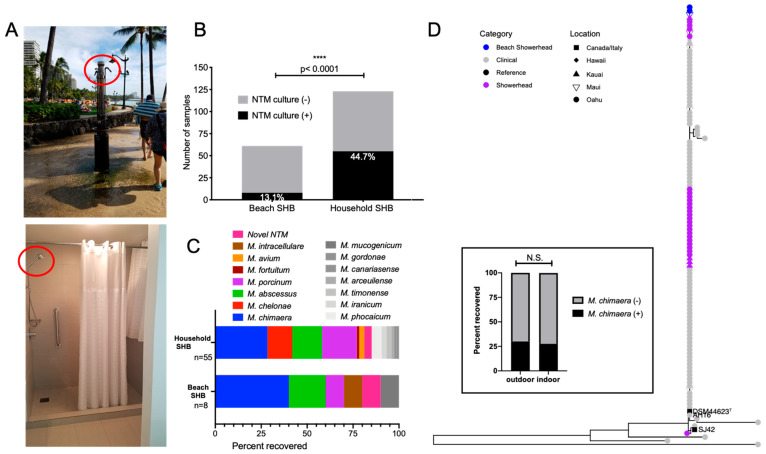
NTM diversity among outdoor beach showerhead biofilms (SHB) compared to indoor household SHB. (**A**) Examples of outdoor beach and indoor household showerheads (red circles, respectively) that were sampled in this study. (**B**) Beach SHB (n = 8/61) were less likely to be NTM culture positive than indoor SHB (n = 55/123). (**C**) Breakdown of the NTM species identified between outdoor beach SHB compared to indoor household SHB. (**D**) Inset box: No significant difference between *M. chimaera* recovery from either SHB type. N.S. = not significant. Phylogenetic analyses of respiratory and environmental SHB *M. chimaera* isolates based on *rpoB* gene sequencing.

Indoor household SHB are known niches for environmental NTM [8,24,25]; however, the prevalence of NTM in outdoor SHB is not known. In Hawai’i, outdoor freshwater showers are commonplace and associated with public beaches (Figure 5A). To investigate NTM diversity among the different Hawai’i showerheads, we compared NTM recovery from beach and household SHB. Significantly less NTM were recovered from beach SHB (8/61, 13.1%) compared to household SHB (55/123, 44.7%) (Figure 5B). *M. abscessus* and *M. porcinum* were identified from both types of SHB. *M. chelonae* and *M. avium* were repeatedly recovered from household SHB, but were absent from beach SHB; whereas *M. intracellulare* was only recovered from beach SHB. *M. chimaera* was the most frequently identified NTM from both outdoor and indoor showerheads (Figure 5C); however, it was recovered in equal proportions from either type of SHB (Figure 5D, inset graph). Phylogenetic analyses indicate that in Hawai’i, patients may have equal opportunities to acquire *M. chimaera* from either outdoor and indoor SHB. This is because *rpoB* gene comparisons showed genetically similar *M. chimaera* colonizing both beach and indoor SHB as respiratory *M. chimaera* isolates (Figure 5D).

## 4. Discussion

In this study, we applied the combination of environmental sampling, microbiological selection, and molecular identification to comprehensively profile the NTM species diversity in outdoor and indoor environments across the Hawaiian Islands, pairing these data with machine learning tools to identify possible epidemiological factors associated with NTM infections.

While we reiterate that the highly ranked variables in Table 3 may not themselves causally influence NTM prevalence in the environment, our models using machine learning revealed a range of environmental, socioeconomic, and demographic factors likely associated with NTM culture status. For example, the probability of finding NTM in Hawai’i outdoor samples was more commonly related to elevation, lowest income quartile, population density, watersheds, and population of Pacific Islanders. We postulate that the relatively high importance of these factors in the outdoor model may be due to differences in how varying socioeconomic groups interact with their environment. For example, in lower socioeconomic areas that are not as populated, it may be more common to find farming and animal husbandry taking place in or near watersheds in mountainous areas at higher elevations. On the other hand, evapotranspiration, elevation, rainfall, median gross rent, and the GINI coefficient were related to the probability of finding NTM in indoor samples. Evapotranspiration has already been associated with high-risk NTM areas in other studies [26]. Typically in cities, evapotranspiration rates are lower because there are less tress and minimal areas of large-scale vegetation [27]. Since our models cannot predict directionality, its possible that lower evapotranspiration rates influence NTM in indoor environments within Hawai’i cities of ranging rental prices and income levels during the wet and dry seasons. As in the case of Honolulu and Waikiki, these cities are typically also found at lower elevations near sea level. Other reports have shown increased county-level risk for NTM PD including population density, median household income, proportion of area as surface water, mean daily evapotranspiration, and greater precipitation associated with NTM PD cases [26]. NTM infections have also been suggested to occur more frequently in wetter climates—supported by the coastal patterns and studies comparing wet and dry regions within the same state [28,29]. Finally, while not a parameter studied here, the sugar and pineapple plantations and agriculture of Kaua’i, O’ahu, Maui, and Hawai’i Island have had significant and differential roles on interisland economies. It would be prudent to assess their possible influence and other similar economic drivers on the distribution of NTM across the islands in future studies.

*M. chimaera*, a species of MAC, represented ~45% of the NTM species identified from respiratory samples from Hawai’i and was the predominant NTM in the Hawai’i environment using partial *rpoB* gene sequencing, data that supports our prior work [8]. *M. chimaera* was identified across all four islands sampled, isolated from all eight types of different water biofilm sources sampled including freshwater streams (Figure 3C), but showed a clear preferable niche in O’ahu indoor showerhead biofilm samples and was not isolated from any outdoor samples collected from Maui or Hawai’i Island (Figure 3B). These data and other collective data from this study (Table 4) further support indoor water biofilms as high exposure points for environmental NTM for patients in Hawai’i, particularly on O’ahu. In addition, phylogenetic analyses indicate a high degree of genetic similarity between respiratory and environmental *M. chimaera* isolates (Figure 5D). This relative genetic homogeneity of *M. chimaera* suggests that predominant *M. chimaera* genotypes are members of a lineage that has recently come to prominence.

While the underlying factors that facilitate *M. chimaera* survival in freshwater and natural areas of Hawai’i is not known, there are likely a variety of contributing factors. In our prior work, we have elucidated potential factors that modulate *M. chimaera* growth in vitro. Specifically, incubation of an environmental *M. chimaera* isolate from Hawai’i with birnessite, a manganese-containing mineral and gibbsite, a mineral form of aluminum hydroxide, both of which are common in Hawai’i soil, significantly reduced *M. chimaera* growth in vitro [30]. However, *M. chimaera* growth remained unchanged in the presence of iron-containing minerals such as hematite, magnetite, and maghemite after 96 h in culture [30], suggesting particular soil minerals influence *M. chimaera* in the environment. The water-associated factors that drive the presence or absence of *M. chimaera* remain to be elucidated, but may include the propensity of *M. chimaera* to form biofilms in man-made instruments as highlighted by the associated heater-cooler outbreaks or other exogenous surfaces [31,32].

In addition to *M. chimaera, M. abscessus, M. chelonae,* and *M. porcinum* were the other most common clinically relevant NTM identified in the environmental samples tested in this study (Figure 3A). Generally, it’s suggested that recovery of *M. abscessus* from the environment remains rare, having been identified in 1–2 samples in various multi-sample studies from South Africa, Taiwan, and the Netherlands [13,33,34,35], but identified with higher frequency (8%) in a potable water study from Australia [36]. Among the 545 environmental samples tested here, *M. abscessus* was identified from 12.9% of all samples tested and was more common in indoor than outdoor samples and routinely recovered from clinical samples (Figure 1) [37]. Thus, the frequency of recovery of *M. abscessus* from the Hawai’i environment is a significant finding and complements our prior work (8).

As another species of MAC, *Mycobacterium avium* subsp. *avium* is also a highly significant NTM responsible for chronic PD in humans [38]. *M. avium* has been recovered from bathtub inlets in Japan [39], showerhead biofilms, soil, dust in Germany [40,41] as well as community and household water in Pennsylvania [42]. However, *M. avium* was rare in the environmental Hawai’i samples tested, but when isolated, it was most often recovered from freshwater streams on O’ahu (Figure 4D). Similar results have been reported from surface river water in Japan [39]. The apparent inverse abundance of *M. chimaera* and *M. avium* in Hawai’i compared to other geographic areas suggest different environmental drivers contribute to NTM species diversity, and their recovery in environmental sampling is reflected in clinical samples, consistent with environmental exposure and acquisition of infections.

Table 4 and Table 5 revealed notable trends regarding NTM recovery from Hawai’i environmental samples. Of the natural sites studied, moss and lava rock were generally devoid of NTM including *M. chelonae*. NTM recovery rates from O’ahu outdoor samples was 12.7% compared to 33.9% of the outdoor samples collected on Maui. Interestingly, the inverse was observed for indoor samples where 54.1% of the O’ahu samples were NTM culture positive compared to 27.3% of the Maui indoor samples. Despite the small number of streams sampled, PD-disease causing NTM were recovered from 8/10 (80%) O’ahu streams compared to 1/11 (9%) Maui streams sampled. The predominance of NTM in the O’ahu environment may be related to population density, urban living, traffic, predominance of household municipal plumbing, freshwater quality, or factors that have not yet been discovered. Again, future studies should investigate these potential factors.

To our knowledge, we are the first to report that outdoor showers such as those found frequently in and around public beaches (Figure 5A) harbor significantly less NTM compared to indoor showerhead biofilms (Figure 5B), suggesting not all showerheads contain an abundance of NTM and NTM prevalence depends on where the showerhead is located. There is likely an assortment of reasons for the scarcity of NTM in beach showerheads, including the constant exposure to outdoor elements (e.g., sun, wind, rain), lack of heated water running through pipes, close proximity to salt water and salt in the air, as well as increased and consistent exposure to the sun and UV radiation [43,44,45]. Of interest, the fairly equivalent recovery of *M. chimaera* from both outdoor and indoor showerheads (Figure 5D, inset) and their genomic similarity in *rpoB* gene sequences compared to respiratory isolates indicate *M. chimaera* infections may be acquired from both outdoor and indoor exposures.

Overall, our data reinforce the outdoor environment as areas of lower exposure for clinically relevant NTM in Hawai’i compared to indoor environments. While NTM PD remains a relatively rare disease, the financial costs associated with clinical treatment continues to grow substantially with Hawai’i showing the highest prevalence of NTM PD cases at 398 per 100,000 population [46]. Despite its significance as a public health challenge, mandatory reporting of NTM PD to public health agencies has not gained momentum because NTM detection could represent colonizing or contaminating organisms [47]. Monitoring NTM PD trends and consistent surveillance in particular geographic hot spots like Hawai’i would significantly improve our understanding of disease trends and help to contextualize our environmental findings to clinical care and NTM PD case rates across each island. We continue to elucidate and study the critical drivers that facilitate NTM prevalence in the environment and advocate for future work to continue to untangle the biological relationships between the environment, host, and NTM in the context of global pulmonary infections.

## Figures and Tables

**Table 1:** Types and number of outdoor and indoor biofilms sampled.

Outdoor (*n* = 179)	Indoor (*n* = 366)
Outdoor (beach) showerheads (*n* = 61)	Showerheads (*n* = 123)
Water fountain (*n* = 24)	Kitchen sink faucets (*n* = 84)
Outdoor faucets (*n* = 9)	Bathroom sink faucets (*n* = 154)
Ice machines (*n* = 12)	Water dispensers (*n* = 5)
Swimming pools (*n* = 12)	
Freshwater streams (*n* = 28) Sphagnum moss (*n* = 26)	
Lava rock (*n* = 7)	

**Table 2:** SNP variation between *rpoB* sequences from 119 showerhead and clinical *M. chimaera* isolates.

Category	Total Isolates (*n*=119)	Beach Showerheads (Mean SNPs ± SD)	Indoor Showerheads (Mean SNPs ± SD)	Clinical Respiratory (Mean SNPs ± SD)
Beach Showerheads	3	0 SNPs ± 0		
Indoor Showerheads	29	0 SNPs ± 0	0 SNPs ± 0	
Clinical Respiratory	87	0 SNPs ± 8.62	1.72 SNPs ± 8.62	3.4 SNPs ± 11.99

**Table 3:** Variance importance ranks of geospatial layers calculated from the machine learning method applied separately to outdoor and indoor samples.

Rank:	Outdoor:	Indoor:
1	Elevation	Evapotranspiration
2	Lowest income quintile	Elevation
3	Population	Rainfall
4	Watershed area	Median gross rent
5	Population of Pacific Islanders	GINI coefficient
6	GINI coefficient	Land development indicator
7	Median gross rent	Watershed area
8	Rainfall	Population with multiracial ancestry
9	Rural population	Soil water infiltration rate
10	Population with multiracial ancestry	Population Pacific Islander
11	Evapotranspiration	Lowest income quintile
12	Soil water infiltration rate	Population on public assistance
13	Land development indicator	Soil pH
14	Soil organic matter	Relative geologic age
15	Soil cation-exchange capacity	Relative humidity
16	Soil shrink-swell capacity	Surface temperature
17	Urban population	Urban population
18	Relative geologic age	Soil cation-exchange capacity
19	Relative humidity	Population
20	Soil pH	Soil organic matter

**Table 4:** NTM recovery per island between outdoor and indoor samples.

	Outdoor Samples		Indoor Samples		
	Total (*n*=)	% NTM Culture Positive	Total (*n*=)	% NTM Culture Positive	*p*-Value
Kaua’i	21	23.8% (5)	30	43.3% (13)	not significant
O’ahu	71	12.7% (9)	146	54.1% (79)	<0.0001
Maui	56	33.9% (19)	88	27.3% (24)	not significant
Hawai’i	31	22.6% (7)	102	34.3% (35)	not significant
**Totals**	**179**	**22.3% (40)**	**366**	**41.3% (151)**	<0.0001

**Table 5:** (**a**) Types of natural sites sampled and the proportion that were NTM culture positive; (**b**) Species of NTM identified from culture positive freshwater streams and moss.

**(a)**					
	**Freshwater Streams**	**Moss**		**Lava Rock**	
*Kaua’i*	0/3 (0%)	Not sampled		Not sampled	
*O’ahu*	8/10 (80%)	0/2 (0%)		Not sampled	
*Maui*	1/11 (9%)	2/19 (10.5%)		0/3 (0%)	
*Hawai’i Island*	0/4 (0%)	1/5 (20%)		0/4 (0%)	
***Total***	**9/28 (32.1%)**	**3/26 (11.5%)**		**0/7 (0%)**	
**(b)**					
**Island:**	**Freshwater Stream:**	**NTM Species Identified from Freshwater Streams:**	**Island:**	**Moss:**	**NTM Species Identified from Moss:**
O’ahu	Stream 1	*M. avium*	Maui	Moss 1	*M. gordonae;* *M. iranicum;* *M. mucogen* *icum*
	Stream 2	*M. chelonae*		Moss 2	Novel NTM
	Stream 3	*M. avium;* *M. chimaera*	Hawai’i Island	Moss 1	*M. rhodesiae*
	Stream 4	*M. abscessus*			
	Stream 5	*M. avium;* *M. timonese*			
	Stream 6	*M. chelonae;* *M. intracellulare*			
	Stream 7	*M. chelonae;* *M. chimaera*			
	Stream 8	*M. chelonae*			
Maui	Stream 1	Novel NTM			

## Data Availability

The new sequence data presented in this study are openly available in NCBI Genbank under the following accession numbers: MW421308-MW421408. Previously published sequences by authors used in this study are also available in NCBI Genbank under the following accession numbers: KU128606-KU128608, KU128610, KU128612, KU128613, KU128618, KU128625, KU128627, KU128653, KU128656, KU128665, KU128666, KU128672, KU128675-KU128681.
